# A First NGS Investigation Suggests No Association Between Viruses and Canine Cancers

**DOI:** 10.3389/fvets.2020.00365

**Published:** 2020-07-17

**Authors:** Diana Giannuzzi, Luca Aresu

**Affiliations:** ^1^Department of Comparative Biomedicine and Food Science, University of Padua, Legnaro, Italy; ^2^Department of Veterinary Science, University of Turin, Grugliasco, Italy

**Keywords:** animal model, canine tumors, NGS, oncogenesis, viral integration

## Abstract

Approximately 10–15% of worldwide human cancers are attributable to viral infection. When operating as carcinogenic elements, viruses may act with various mechanisms, but the most important is represented by viral integration into the host genome, causing chromosome instability, genomic mutations, and aberrations. In canine species, few reports have described an association between viral integration and canine cancers, but more comprehensive studies are needed. The advancement of next-generation sequencing and the cost reduction have resulted in a progressive increasing of sequencing data in veterinary oncology offering an opportunity to study virome in canine cancers. In this study, we have performed viral detection and integration analyses using VirusFinder2 software tool on available whole-genome and whole-exome sequencing data of different canine cancers. Several viral sequences were detected in lymphomas, hemangiosarcomas, melanomas, and osteosarcomas, but no reliable integration sites were identified. Even if with some limitations such as the depth and type of sequencing, a restricted number of available nonhuman genomes software, and a limited knowledge on endogenous retroviruses in the canine genome, results are compelling. However, further experiments are needed, and similarly to feline species, dedicated analysis tools for the identification of viral integration sites in canine cancers are required.

## Introduction

An estimated 10–15% of all human cancers are attributed to viruses, including papillomavirus (PV), hepatitis B virus (HBV), Epstein–Barr virus (EBV), human T-lymphotropic virus, Kaposi sarcoma–associated herpesvirus, and Merkel cell polyomavirus (1–6). Although individual tumor viruses exert their oncogenic effects in different ways, a common feature is represented by the integration of the viral genome into the host, causing gene mutations, chromosome instability, and genomic aberrations (7, 8). In canine species, only few reports have pinpointed an association between viruses and tumors, and canine PV integration was described both in squamous cell carcinoma and transmissible venereal tumor (9–12). Although the presence alone of viral sequences in tumor genome is insufficient to prove an oncogenic role, viral integration may represent the first hallmark of the malignant transformation. Indeed, interplay between viral variants and proto-oncogenes conferring malignancy needs to be proved. This is the case of gammaretrovirus feline leukemia virus (FeLV) that is widely recognized to be oncogenic, predisposing cats to lymphoma, and fibrosarcoma (13). The pathogenic mechanisms of FeLV influencing lymphoma transformation were previously identified and include common integration sites nearby proto-oncogenes, resulting in insertional mutagenesis (14). Conversely, the association of feline immunodeficiency virus (FIV) in feline lymphoma was reported as a rare event (15, 16), and the pathogenetic role is scarcely known. Furthermore, FIV-related malignant transformation seems to imply different mechanisms from those employed by FeLV, and a causal association has been described only when provirus integration in tumor DNA is monoclonal (17).

The advent of next-generation sequencing (NGS) has caused a tremendous impact both on basic and clinical oncology research. Next-generation sequencing analysis of tumor genetic changes, transcriptomes, and epigenomes is driving biomarker discovery for cancer diagnostic and therapy, but it also opens opportunities for the identification of new associations between tumors and viruses (2, 8, 18). Oncoviral profiles are obtained from cancer genome sequencing data, and several algorithms have been developed to identify viral integration sites (19–23), but only a few are available for nonhuman host genomes (19, 24). In any case, the computational approaches are very similar and based on the identification of one or more reads containing a portion of nucleic acid sequences uniquely mapping to a viral genome integrated within tumoral DNA. As costs become less prohibitive, and methods simpler, veterinary researchers are choosing NGS over microarrays to study canine tumors, and the recent studies published on this topic provide an unprecedented opportunity to study canine virome.

Considering these premises, our study aims at (i) detecting the presence of viruses of arbitrary types in NGS data from several canine tumor histotypes; (ii) identifying virus integration sites as long as viruses merge into the genome and the sequencing captures the insertion sites; and (iii) evaluating the potential pathogenic role of the viral presence/integration.

## Methods

First, we have performed a systematic review to retrieve the current literature where NGS technology was applied to canine tumors, and if publicly available, we downloaded FASTQ files of paired-end whole-exome sequencing (WES) and whole-genome sequencing (WGS) using the SRA toolkit (https://www.ncbi.nlm.nih.gov/sra/docs/toolkitsoft/). The dataset included 107 lymphomas (25), three transitional cell carcinomas (TCCs) of the urinary bladder (26), 20 splenic hemangiosarcomas (27), 11 malignant melanomas (28), 18 acanthomatous ameloblastomas (29), 46 osteosarcomas (30), 22 gliomas (31), 20 mammary tumors (32), and 28 mast cell tumors (33) (Table S1). The mean sequencing coverage of each dataset is reported in Table S2. The viral detection, integration, and mutation identification were performed using VirusFinder2 software tool implemented by virus integration site detection through reference sequence customization (VERSE) (19, 24). Briefly, VirusFinder2 integrates a mixed strategy of NGS reads alignment. First, raw reads are aligned to the host reference genome, and then the unmapped are aligned to a hybrid reference genome for virus detection and integration (34). In this study, all the sequences were aligned to the dog genome (CanFam3.1, Sept. 2011, Ensembl release 96). The virus genomes were obtained from RINS package (35) and National Center for Biotechnology Information (NCBI) virus database (https://www.ncbi.nlm.nih.gov/labs/virus/vssi/#/). In details, RINS package includes 32,102 viral genomes derived from all the classes in GenBank (36) and International Committee on Taxonomy of Viruses retrieved through the NCBI. We included all partial and complete sequences (*n* = 16,394) of known viruses having *Canis lupus familiaris* as a putative host from the NCBI virus database. The configuration file was set with default parameters, except for “sensitivity_level = 2” and “min_contig length = 200,” which were modified to reach a higher sensitivity during virus detection. The detailed list of third-party tools used by VirusFinder2 and an example of configuration file are provided in Supplementary Methods.

## Results

After the preprocessing step, the pipeline of VirusFinder2 follows a two-step procedure: (1) virus detection and (2) virus integration site detection. The virus detection did not retrieve viral sequences in TCCs and ameloblastomas. Conversely, several putative viral sequences were aligned against host unmapped sequences with high identity (>85%) in mammary tumors (*n* = 4), gliomas (*n* = 1), mast cell tumors (*n* = 7), lymphomas (*n* = 8), hemangiosarcomas (*n* = 6), melanomas (*n* = 11), and osteosarcomas (*n* = 27). By definition, the identity indicates similarity between host unmapped and viral sequences. Further, the data were manually checked for common bacteria and bacteriophage sequences and when identified were excluded as result of inaccurate annotation (37). Also, NGS computational artifacts and common viral contaminants including *Choristoneura occidentalis* granulovirus and *Paramecium bulsaria chlorella* virus were discarded (38, 39). The host genome unmapped reads fallen on contigs lower than 1,000 were excluded.

After this filtering process, all the candidate viruses in gliomas, hemangiosarcomas, and mammary tumors resulted false positives. Instead, 1 lymphoma, 7 mast cell tumors, and 12 osteosarcomas retained several candidate viruses. The top-ranking virus candidate was investigated for each tumor based on percentage of identity, length of the contigs, and number of reads fallen on contigs (Table S3). Then, the viral integration was tested, but only three osteosarcomas showed a positive result. Viruses identified were Meleagrid herpesvirus (HV) 1, Gallid HV 3, and Human HV 7 (Table 1), but a low confidence was assessed.

**Table 1:** List of virus integration sites in all the cancer samples.

**Sample (1)**	**Chr (1)**	**Position (1)**	**Virus (2)**	**Chr (2)**	**Position (2)**	**No. support reads**	**Confidence**
						**(pair + softclip)**	
SRR8691727	chr12	72,497,897	AF282130.1__Meleagrid_herpesvirus_1	Viral genome	117,487–117,676	31 + 9	Low
SRR8691785	chr12	72,497,897	NC_002577.1__Gallid_herpesvirus_3	Viral genome	133,794–134,005	73 + 18	Low
SRR8691786	chr12	72,497,897	NC_001716.2__Human_herpesvirus_7	Viral genome	143,544–143,960	27 + 11	Low

*Chr, chromosome; 1, attributes related to the cancer sample; 2, attributes related to the virus; pair + softclip, the word pair means the number of paired-end reads whose one end mapped to the host genome and another aligned to the integrated virus sequence. The word softclip means the number of reads that actually harbor the integration breakpoint within themselves. Softclip is the total soft-clipped reads that map to the host reference genome plus the total soft-clipped reads that aligned to the virus sequence; confidence: if the number of soft-clipped reads is sufficient to support an accurate characterization of the locus*.

Because several herpesviruses were retrieved among the top-ranking viruses, we manually checked the correspondences. All the sequences aligned to unspecific homopolymer-rich sequences, confirming previously published data reporting that herpesviruses harbor telomeric repeats almost identical to mammalian telomeres (40–42). Also, a retrovirus was included in the list, but the sequence resulted in an long terminal repeat (LTR)-rich sequence, likely corresponding to proviral DNA or endogenous retroviruses (ERVs).

Last, to get insight into the putative viral sequences of lymphoma, mast cell tumor, and osteosarcoma samples, the GenBank nonredundant database (ftp://ftp.ncbi.nlm.nih.gov/blast/db/FASTA/) was interrogated. Correspondences were obtained only in osteosarcoma, mainly matching with *Macrostomum lignano* and *Branchiostoma floridae*, although identity was overall low (<55%).

## Discussion

In recent years, a significant amount of NGS data has been produced in dogs, but no studies were conducted to investigate virome in cancers. Also, several algorithms have been developed for NGS investigation of virus–host interactions in human species. In this study, we selected VirusFinder2, which resulted the most performant software by far. This was similarly demonstrated in a previous study comparing methods for the identification of viral integrations in genomes (34). Indeed, VirusFinder2 was tested on several human cancer genomes (19, 24) and showed the highest sensitivity and precision in high-coverage datasets (>60×) compared to other software able to handle multiple-virus integration. In our dataset, the tumors were sequenced with a mean coverage higher than 60×, with the exception of the TCC and melanoma WGS samples (Table S2). Further, VirusFinder2 integrates nonhuman genomes representing a significant advantage in veterinary medicine.

Given these premises, in our study, we tested a large WGS and WES canine tumors dataset, but none of the detected virus sequences and viral integration sites were reliable, and this is determined by the low confidence of the integration results. Indeed, all the detected viruses do not infect the canine species, and the candidate sequences were mostly unspecific homopolymer-rich sequences of herpesviruses or LTR-rich sequences of retroviruses. Few sequences also aligned with genomes of marine organisms, suggesting a contamination of the tumor samples.

Interestingly, several sequences were suspected to correspond to ERVs. Endogenous retroviruses show a ubiquitous presence in vertebrate genomes comprising up to 5–8% of the human genome. While the functional effects of ERVs are well-established, and the vast majority of ERV insertions are fixed across individuals, the potential contribution to phenotypic variation is still unknown in dog. Also, degeneration due to the accumulation of mutations can occur during life (43, 44). In cats infected by FeLV, a recombination between endogenous and exogenous viruses can generate strains with specific pathogenic capabilities (45). The canine genome displays only a low percentage (~0.15%) of ERVs compared to other mammals (46, 47), and this is mainly related to a low number of repetitive elements in the genome (48). Despite hematological malignancies are frequent in dogs, and retroviral particles have been reported both in lymphomas and cell lines (49, 50), no exogenous retroviruses have been identified in dogs or any other canid so far. The lack of exogenous retroviruses and the low number of ERVs are quite surprising mainly because canine cells are known to enable the replication of noncanid exogenous retroviruses, and the canine genome lacks the functional retroviral restriction factor TRIM5α (51). Recent studies have proposed that the expansion of an endogenous gammaretroviral lineage might originate from an infection in canid ancestors, thereby supporting the potential existence of canine exogenous retroviruses (46, 52). Further studies are needed to confirm these findings.

A partial explanation for the negative results obtained here may be the selected dataset that comprised mainly WES data, implying that sequencing targets covered only exonic regions. Although retroviruses can integrate randomly or in transcription units, introns and exons of active genes (5), the majority of retroviruses, PVs, HBV, and EBV, favor integration into promoters and transcription start sites of active genes (53–55) that are rarely sequenced in WES. Here, the integration sites were retrieved only in WGS samples.

Finally, although this is not the case, the presence alone of viral sequences in tumor genome is insufficient to prove an oncogenic role. In humans, several viruses are widely distributed in the population such as EBV and human HV 6, but not causing malignancies. A more complicated aspect to solve is the discrimination of the time point at which viral infections may have arisen: if infections occur after tumor growth, then they have no contribution to tumorigenesis. Unfortunately, we could not expand this aspect considering the negative results.

In conclusion, we have analyzed WES and WGS data derived from different canine tumor histotypes to detect potential viral integration sites. Even if previous lines of evidence have hypothesized a role of viruses in canine lymphoma (56), we could not confirm this hypothesis. However, similarly to feline species (57), studies dedicated to identify viral integration sites in canine cancers are needed, and transmissible venereal tumor and squamous carcinoma represent excellent models to further test VirusFinder2.

## Data Availability Statement

Publicly available datasets were analyzed in this study. This data can be found here: PRJNA389294, PRJNA478811, PRJNA417727, PRJNA525883, PRJNA516699, PRJNA339175, PRJNA247493, and PRJNA247491.

## Author Contributions

DG and LA contributed conception and design of the study; DG selected the datasets and performed the computational analyses; DG and LA wrote the manuscript; LA edited the manuscript. All authors contributed to the article and approved the submitted version.

## Conflict of Interest

The authors declare that the research was conducted in the absence of any commercial or financial relationships that could be construed as a potential conflict of interest.
