# Supplementary material for: A First NGS Investigation Suggests No Association Between Viruses and Canine Cancers
Source: Front Vet Sci. 2020 Jul 17;7:365. doi: 10.3389/fvets.2020.00365 (PMC7380080; doi:10.3389/fvets.2020.00365)
Supplement: Supplementary file 4 [file Data_Sheet_1.DOCX]

Supplementary Material

**Supplementary Methods.** List of third-party tools used by VirusFinder2 and an example of configuration file.

**Supplementary Table S1.** List of the WGS and WES data of canine tumor samples downloaded from NCBI Sequence Read Archive (SRA) repository and used in the present study. The worksheet is organized as follows: Column A: bioproject ID; column B:sample ID; column C: type of sequencing; column D: sample tissue; column E: type of disease; columns F, G, H: breed, sex and age of the dog.

**Supplementary Table S2.** List of the average sequencing coverage of the samples considered in this study. The worksheet is organized as follows: Column A: tumor histotype; column B: reference of the considered dataset; column C: type of sequencing; column D: mean coverage of samples employed in the study.

**Supplementary Table S3.** List of the top-ranking detected viruses in publicly available sequenced genomes of canine cancers. For each sample is reported the disease, the top-ranking virus, the identity between the virus and host unmapped reads, the candidate sequence length (bp), the number of reads fallen on the candidate sequence, the candidate sequence Trinity reference ID, the candidate sequence FASTA and the organism and the E-value reported in the blast+ alignment against the Genbank’s non-redundant database.
