# Supplementary material for: A First NGS Investigation Suggests No Association Between Viruses and Canine Cancers
Source: Front Vet Sci. 2020 Jul 17;7:365. doi: 10.3389/fvets.2020.00365 (PMC7380080; doi:10.3389/fvets.2020.00365)
Supplement: Supplementary file 5 [file Data_Sheet_2.DOCX]

**Supplementary Methods**

1.Third party tools used in VirusFinder2.

**Alignment tools**

BLAST+ version 2.2.26+

BLAT1 version v.34

Bowtie2 version 2

**Reference correction tool**

iCORN version 10.97

**Structural variant calling**

CREST version 11.1

SVDetect version r0.8

**Realignment tool**

GATK version 2.4-9

**Alignment processing tool**

SAMtools version 0.1.18

**De novo assembly tool**

Trinity version 2012-06-08

2. Example of configuration file.

**#Input files**

#alignment_file=

fastq1=/home/xxx/Virus_integration/Samples/*_1.fastq.gz

fastq2=/home/xxx/Virus_integration/Samples/*_2.fastq.gz

detect_integration=yes

detect_mutation=yes

mailto=xxx@xxx

thread_no=15 *# the number of threads for parallel computing*

**#Paths for thirdy-party tools**

blastn_bin=/usr/bin/blastn

bowtie_bin=/opt/bowtie2-2.2.9/bowtie2

trinity_script=/opt/trinity/Trinity.pl

SVDetect_dir=/opt/SVDetect_r0.8b

**#Reference files (indexed for Bowtie2 and BLAST)**

virus_database =/home/xxx/Virus_integration/Virus/virus.fa

bowtie_index_human =/home/xxx/Virus_integration/Dog_Genome/CanFam3.1.96

blastn_index_human =/home/xxx/Virus_integration/Dog_Genome/CanFam3.1.96

blastn_index_virus =/home/xxx/Virus_integration/Virus/virus

**#Parameters of of virus insertion detection (VERSE algorithm)**

detection_mode= sensitive

*#Possible values: {normal, sensitive}; default value: normal. If not specified, VirusFinder runs in normal detection mode.*

flank_region_size=4000

*#Suggested values: >2000; default: 4000;if detection_mode = normal, it (and ‘sensitivity_level’ below) will be ignored.*

sensitivity_level=2

*#Suggested values: 1 to 6; default value: 1; greater value means higher sensitivity, and accordingly more computation time.*

**#Parameters of virus detection**

min_contig_length=200

blastn_evalue_thrd=0.05

similarity_thrd=0.8

chop_read_length=25

minIdentity=80
